# Effect of changes in the fractal structure of a littoral zone in the course of lake succession on the abundance, body size sequence and biomass of beetles

**DOI:** 10.7717/peerj.5662

**Published:** 2018-09-26

**Authors:** Joanna Pakulnicka, Andrzej Zawal

**Affiliations:** 1 Department of Ecology and Environmental Protection, Faculty of Biology and Biotechnology, University of Warmia and Mazury Olsztyn, Olsztyn, Poland; 2 Department of Invertebrate Zoology and Limnology, Institute for Research for Biodiversity, Centre of Molecular Biology and Biotechnology, Faculty of Biology, University of Szczecin, Szczecin, Poland

**Keywords:** Water beetles, Lakes, Disharmonic succession, Fractal dimension, Body size

## Abstract

Dystrophic lakes undergo natural disharmonic succession, in the course of which an increasingly complex and diverse, mosaic-like pattern of habitats evolves. In the final seral stage, the most important role is played by a spreading *Sphagnum* mat, which gradually reduces the lake’s open water surface area. Long-term transformations in the primary structure of lakes cause changes in the structure of lake-dwelling fauna assemblages. Knowledge of the succession mechanisms in lake fauna is essential for proper lake management. The use of fractal concepts helps to explain the character of fauna in relation to other aspects of the changing complexity of habitats. Our 12-year-long study into the succession of water beetles has covered habitats of 40 selected lakes which are diverse in terms of the fractal dimension. The taxonomic diversity and density of lake beetles increase parallel to an increase in the fractal dimension. An in-depth analysis of the fractal structure proved to be helpful in explaining the directional changes in fauna induced by the natural succession of lakes. Negative correlations appear between the body size and abundance. An increase in the density of beetles within the higher dimension fractals is counterbalanced by a change in the size of individual organisms. As a result, the biomass is constant, regardless of the fractal dimension.

## Introduction

Ecological succession is a natural course of events that occurs in lakes (Kajak, 1998). Lake succession manifests itself in the growth of macrophytes along lake shores, which initially create increasingly diverse and compact communities (McFarland, Carse & Sandin, 2009; Drinan et al., 2013; Beadle, Brown & Holden, 2015; Šiling & Urbanič, 2016; Stryjecki et al., 2017), only to have the littoral zone ultimately dominated by a single species. If the succession is disharmonic, the dominant species is *Sphagnum* sp. (Kajak, 1998), whereas in lakes undergoing harmonic succession the prevalent species are most often *Phragmites australis* (Ciecierska, 2008; Pakulnicka, Górski & Bielecki, 2015b), and less frequently *Carex* sp., *Sparganium* sp. or *Acorus* sp. Many hydrobiologists emphasize the important role of the littoral zone in the secondary production of lakes and as a zone having the highest species richness and density of macroinvertebrates (Cremona, Planas & Lucotte, 2008; Timm & Möls, 2012; Mieczan et al., 2014; Płaska & Mieczan, 2018). The littoral zone is also considered to be the most sensitive part of a lake, and its character provides evidence on the ecological condition of the whole lake (Czachorowski, 1998; Šiling & Urbanič, 2016).

The succession of lakes entails changes in the lake-dwelling fauna (Kowalik, 1968; Pakulnicka & Bartnik, 1999; Ranta, 1985; Kordylas, 1990; Lundkvist, Landin & Milberg, 2001; Pakulnicka & Zawal, 2007). This problem is rarely raised in research, and when it is, it most often consists of brief studies into zooplankton (e.g., Demetraki-Paleolog, 2012; Kuczyńska-Kippen, 2008; Jasser, 1997; Lepistö & Saura, 1998; Odland & Del Moral, 2002; Fiłoc & Kupryjanowicz, 2015). More detailed investigations pertaining to changes in lake fauna during consecutive seral stages are conducted on anthropogenic rather than natural ecosystems (Pakulnicka, 2008; Bloechl et al., 2010; Buczyński, 2015; Pakulnicka, Górski & Bielecki, 2015b). Hence, despite the wealth of references, our knowledge of the succession mechanisms in lake fauna is modest and fragmentary, even though it is extremely important for developing proper lake management practice (Šiling & Urbanič, 2016; Shadrin et al., 2016).

Another challenge for researchers is to find an adequate measure for the determination of changes in fauna induced by the succession of water bodies. Hydrobiologists most often draw attention to changes in the abundance and species richness while comparing small groups of lakes with different trophic states (Kowalik, 1968; Ranta, 1985; Kordylas, 1990; Lundkvist, Landin & Milberg, 2001; Pakulnicka & Zawal, 2007; Soldán et al., 2012; Barndt, 2012; Drinan et al., 2013; Pakulnicka et al., 2013; Baars et al., 2014; Beadle, Brown & Holden, 2015). A measure that has been gaining popularity over the last twenty years consists of an analysis of quantitative relations between components (generalists and diversified specialists) distinguished on the basis of their affinity towards specific habitat conditions (e.g. Kowalik, 1968; Kordylas, 1990; Czachorowski, 1998; Pakulnicka & Zawal, 2007; Płaska & Tarkowska-Kukuryk, 2014; Pakulnicka et al., 2016a; Pakulnicka et al., 2016b; Płaska et al., 2016; Stryjecki et al., 2017). However, the results obtained from this approach are discrepant and therefore not highly reliable, often because of a small number of samples or analysed objects.

An important addition to research consists of analyses based on biometric measurements, found also in studies on small water organisms, including beetles (Jeffries, 1993; McAbendroth et al., 2005; Ulrich, 2007; Vamosi, Naydani & Vamosi, 2007; Cremona, Planas & Lucotte, 2008; Scheffer et al., 2015; Scheffer & Van Nes, 2006; Tokeshi & Arakaki, 2012; Désamoré et al., 2018). In recent years, biometric measures have become a tool applied in evolutionary ecology. Some hydrobiologists, e.g., Scheffer & Van Nes (2006) and Scheffer et al. (2015), analyse the structure of body sizes of organisms in specific communities from the viewpoint of coevolution of concurrent species. Others, e.g., Désamoré et al. (2018), search for relationships between the evolution of a body size and the environment, as well as the diversification of species in various water habitats.

Data regarding the body mass and body size of macroinvertebrates often appear in the context of studies on a fractal structure, a notion which still awaits a better understanding (Jeffries, 1993; Tokeshi & Arakaki, 2012; Barnes, Vaughan & Ormerod, 2013), and a fractal dimension, a borrowing from mathematical sciences (Mandelbrot, 1983). According to Andrejczuk (2014), all landscapes with inner diversity demonstrate fractality, i.e., they are composed of smaller fragments, self-similar fractals, which are self-reproducing duplicators of parameters on a different scale. Thus, a fragment of any system should contain all the system’s characteristics in a nutshell (on a smaller scale). The littoral zone of ecologically young lakes can serve as an example of a linear structure of spatial organisation which demonstrates properties of fractality. However, as the succession progresses, the shoreline becomes more diverse and turns into a multifractal system, i.e., heterogeneous, composed of fragments (separate subsets) with different, local characteristics, each presenting self-similar properties (Miałdun, 2010; Miałdun & Ostrowski, 2010). Thus, similar fragments (fractals) of the littoral zone of different objects should be characterised by similar habitat traits, offering the same niches to the organisms which populate them. A convenient and valuable object in studies on the fractal structure are dystrophic lakes, in which various habitats (fragments of the littoral zone) appear during the course of succession: from a plant-free zone to zones overgrown with macrophyte communities of various compactness to a compact *Sphagnum* mat with small pools.

In our study we looked at water beetles because they are particularly numerous organisms in the littoral zone as well as being very sensitive and responsive to any unfavourable changes in the environment (Eyre, Foster & Foster, 1992; Foster & Eyre, 1992; Corbet et al., 1980; Winfield Fairchild, Faulds & Matta, 2000; Bosi, 2001; Menetrey et al., 2005; McFarland, Carse & Sandin, 2009; Gioria, Bacaro & Feehan, 2010a; Gioria et al., 2010b; Yee, 2014). Furthermore, many species are predators, which defines their important role as organisms regulating the abundance and species richness of concurrent taxa (Yee, 2014; Frelik & Pakulnicka, 2015; Frelik & Pakulnicka, 2015; Perissinotto, Bird & Bilton, 2016; Miller & Bergsten, 2016; Płaska & Mieczan, 2018). Water beetles penetrate both the ground and the water column, so according to McAbendroth et al. (2005), their ecological niche is three-dimensional. Consequently, the fractal dimension of the habitats they occupy is within the range of 2 < *D* < 3 (increasing from the least to the most compact habitats) (Cremona, Planas & Lucotte, 2008; Tokeshi & Arakaki, 2012).

In the light of the above considerations, our aim has been to investigate whether (1) the distinguished habitats are populated by similar assemblages of organisms with respect to body size and trophic preferences, (2) if there is a relationship between the body mass and abundance of water beetles, (3) if the fractal dimension of a habitat has an effect on the total biomass of beetles, and whether the total biomass of beetles in particular habitats is the same, and finally (4) if there are changes in the sequence of body size of organisms occurring in the course of disharmonic succession.

## Material & Methods

### Study area and field studies

**Figure 1:**
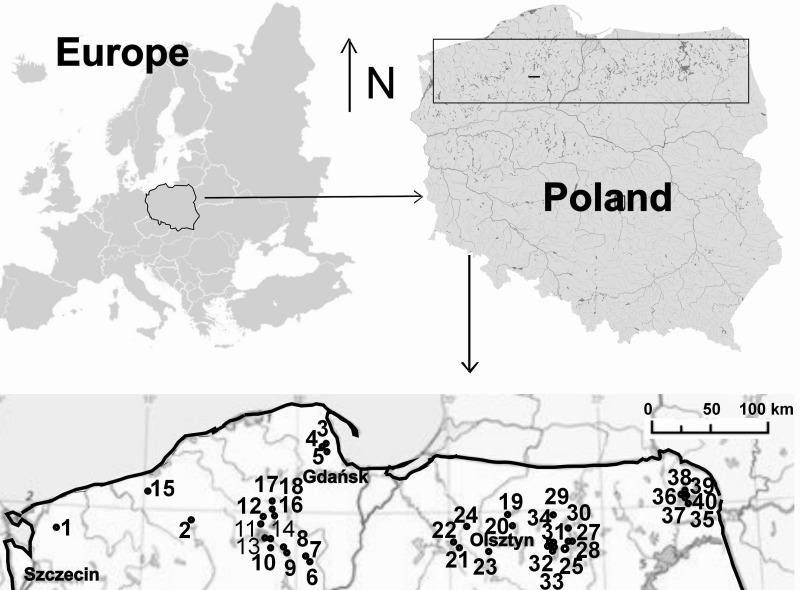
Study area. Location of lakes (1–40): 1, Żabie; 2, Szare; 3, Wygoda; 4, Krypko; 5, Pałsznik; 6, Małe Gacno; 7, Wielkie Gacno; 8, Długie; 9, Moczadło; 10, Sosnówek; 11, Żabionek; 12, Klimontek; 13, Nierybno; 14, Małe Łowne; 15, Czarne; 16, Piecki; 17, Babionek Duży; 18, Babionek Mały; 19, Białe; 20, Kociołek; 21, Żabie; 22, Motylek; 23, Purdka; 24, Jonkowo; 25, Borkowskie; 26, Bobrówko; 27, Gryżewskie; 28, Skarp; 29, Zakręt; 30, Kruczy Stawek; 31, Kruczek Duży; 32, Kruczek Mały; 33, Krucze Oko; 34, Kruczy Staw; 35 Suchar Wielki; 36, Suchar 1 Lake; 37, Suchar 2; 38, Suchar 3; 39, Suchar 4; 40, Suchar 5.

The study covered 40 dystrophic lakes located in northern Poland: the South Baltic Coastland, the West Pomeranian, Olsztyn, Mrągowo Lakelands and the Suwałki Lakeland (Fig. 1, Appendix S1). The lakes chosen for the study were of various surface areas and having a floating peat mat growth belonging to *Sphagnum* sp. that varied in size. The lakes represented various succession stages in a disharmonic series—from oligo- to polyhumic lakes. The oligohumic lakes were inhabited by *Juncus bulbosus*, *Eleocharis palustris*, *Phragmites australis*, *Typha angustifolia*, *Typha latiffolia*, *Sphagnum* sp., *Lobelia dortmanna*, *Isoëtes lacustris*, *Drosera rotundifolia*, *D. anglica*, *D. intermedia*, *Sparganium angustifolium*, *Lycopodiella inundata.* In polyhumic lakes, the dominant species included *Sphagnum* sp., *Oxycoccus quadripelatus*, *Andromeda polifonia*, *Eriophorum vaginatum*, *Calluna vulgaris*, *Erica tetralix*, *Empetrum nigrum*. The lakes were arranged *a priori* according to the seral stages of succession (Bloechl et al., 2010), and the point of reference was assumed to be the coverage of a lake’s surface by a *Sphagnum* mat (0–75%) (Appendix S1). The percentage cover by a *Sphagnum* mat in each lake was calculated in the GIS system, supported by ArcMap 9.3.1 software. For charting the research sites, data made available through Geoportal 2 in the WMS format were employed.

**Figure 2:**
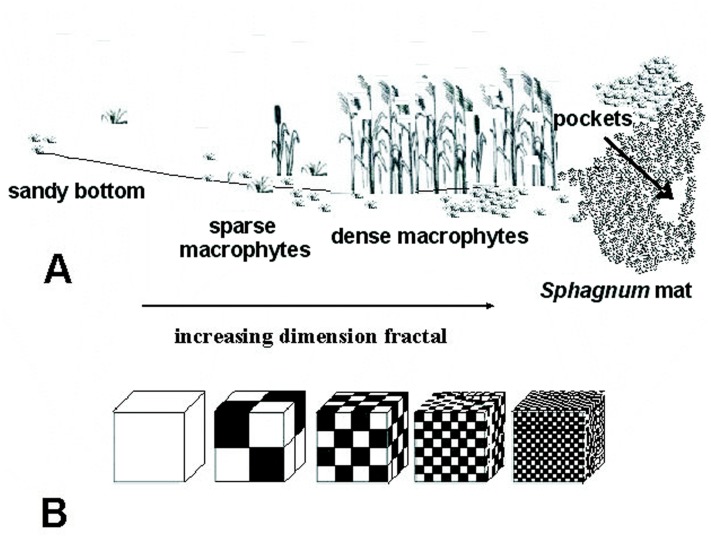
Distinguished habitats in the littoral zone of lakes (A) and hypothetical fractal dimensions (B) (after Tokeshi & Arakaki (2012), modified).

The study was carried out from 2002 to 2014, in spring, summer and autumn. The fauna samples were collected with a dip net from a surface area of about 1 m^2^. In the compacted *Sphagnum* mat environment, a sample comprised 10 subsamples (aggregated afterwards), which were collected using a 0.1 m^2^ strainer. Sampling sites were chosen so as to represent the biggest array of the littoral habitat diversity and areas of individual lakes. Thus, five different littoral components (habitats) were identified: (1) the *Sphagnum* mat, (2) sparse macrophyte zone, (3) dense macrophyte zone, (4) sandy bottom zone and (5) pockets and ponds within a *Sphagnum* mat. The vegetation cover was assessed using Braun-Blanquet (1964) phytosociological records. All lakes were identified with respect to the surface area, extent of the lake’s surface covered with a *Sphagnum* mat, which corresponds to succession stages (1–3), percent share of individual habitats in the littoral zone (1–4) and a seral stage (1–40) (Appendix S1). It was assumed that these habitats, due to different degrees of complexity, represent different fractal dimensions, all within an interval of 2.0 >*D* >3.0 (Tokeshi & Arakaki, 2012). A schematic representation of fractals different in size can be seen in Fig. 2. The smallest size (1) corresponds to the sandy bottom zone, while the largest one (5) is assigned to the *Sphagnum* mat. The intermediate sizes belong to: (2) pockets and ponds within the *Sphagnum* mat, (3) sparse macrophyte zone, and (4) dense macrophyte zone. Field studies were conducted in accordance with field-study-approvals: Certificate of RDOS in Olsztyn (WOPN.070.21.2018.AKI), WNP Certificate (PNE 08-070/ 18) and ZPK Certificate (060.1.2018).

In total, 499 samples were collected. Subsequently, the collected samples were described according to the chosen environmental parameters: habitat (1—*Sphagnum* mat, 2—sparse macrophytes, 3—dense macrophytes, 4—sandy bottom, 5—pockets and ponds within *Sphagnum* mat), place (location) (1–ecotone, 2—pockets and ponds within the *Sphagnum* mat, 3—compacted *Sphagnum* mat), bottom (soil substrate) (1—sand, 2—sand and mud, 3—*Sphagnum*), depth (1—0–10 cm, 2—0–20 cm, 3—20–40 cm, 4—40–60 cm), nympheids (1—none, 2—present), elodeids (1—none, 2—present), (detritus (1—none, 2—scarce and fine, 3—abundant and fine, 4—abundant and coarse), debris (1 –none, 2—present), fractal dimension (1–5), stage—seral stage of succession (1–40).

### Ecological and statistical analyses

Species diversity was calculated using: *S*—number of species, *N*—number of individuals and *D*—percent share. All collected beetles were divided into five classes of different body size (where a body size meant the total length of an organisms), i.e., 1—very small beetles (<3.0 mm), 2—small beetles (3.0–5.0 mm), 3—medium beetles (5.1–10.0 mm), 4—large beetles (10.1–20.0 mm) and 5—very large beetles (>20.0 mm). In addition, the body weight was measured within each of the five classes. The ranges of body weight values of beetles in the distinguished body size classes are shown in Table 1. Three functional groups were distinguished in the trophic structure of beetles: predators (families: Gyrinidae, Dytiscidae and Noteridae), polyphages (Haliplidae) and saprophages (Helophoridae, Hydrophilidae and Hydraenidae) (Appendix S2). Collected adult water beetles were identified using standard identification keys (Galewski, 1990; Galewski & Tranda, 1978; Friday, 1988; Hansen, 1987; Holmen, 1987).

**Table 1:** General description of the lake beetles.

**Body size class**	**Abundance**	**Number of species**	**Body size (mm)**		**Body weight (mg)**	
			**(mean ± SD)**	**min–max**	**mean ± (SD)**	**min–max**
Very small	2,953	32	2.25 ± 0.38	1.30–2.95	5.89 ± 0.41	4.9–6.6
Small	5,640	37	3.47 ± 0.56	3.00–4.90	7.90 ± 0.78	7.10–8.89
Medium	945	26	5.82 ± 1,12	5.00–9.80	38.28 ± 3.76	25.80–42.16
Large	565	24	13.06 ± 2.37	10.5–18.6	234.26 ± 83.16	190.28–315.26
Very large	36	5	31.79 ± 2.09	27.5–35.1	2,228.02 ± 180.4	2,130.2–2,950.1

**Notes.**

Min minimum value max maximum value mean average value SD standard deviation

Because the samples of water beetle fauna were collected several times (taking into account the phenological aspect) from the same lakes and from the habitats distinguished within these lakes, we used a GLM (Generalized Linear Model) for repeated measures ANOVA (Hocking, 1996) to determine the significance of differences in the number of beetles within each functional group in the distinguished habitats, and to identify dependencies between the type of habitats, body size, abundance and biodiversity of Coleoptera. First, we checked the assumptions of normality (the Shapiro–Wilk test) and homogeneity of variances (the Levene’s test), respectively. The GLM repeated measure models were calculated on the basis of Type III sums of squares so as to take the unbalanced design into account. Significant results were tested for pair-wise comparisons by the Tukey’s HSD *post-hoc* tests. Dependent variables (abundance and number of species) were transformed where necessary to fulfil the requirements of parametric tests (Saint-Germain et al., 2007; Cremona, Planas & Lucotte, 2008).

In four separate analyses, we calculated the Pearson’s correlation coefficients (*r*_*p*_) in order to determine correlations between: (1) body size and counts of beetles in the distinguished habitats, (2) cover of the *Sphagnum mat* and counts of beetles, (3) cover of the *Sphagnum mat* and functional groups, and (4) species and environmental variables.

Linear regression analysis was performed to determine the influence of: (1) the body size on the abundance of beetles, (2) fractal dimension on the mean body size on, (3) fractal dimension on the mean weight, and (4) the seral stage of succession on the mean body size. Multidimensional correspondence analysis (MCA) (Clausen, 1998) served to determine dependencies between the abundance of water beetles within the identified body size classes, the share of the distinguished habitats in the littoral zones and in the analysed lakes. MCA has been used in similar studies, e.g., Usseglio-Polatera, Bournaud & Tachet, 2000; Obolewski, Glińska-Lewczuk & Kobus, 2009. The average density of body size classes in a sample was adjusted by the value corresponding to the total contribution of a given littoral zone type in individual lakes, thus giving the weighted average. The analysis included two dimensions, of which the first explained the biggest part of the general chi-squared statistics (% of inertia), whereas the inclusion of the other dimension increased the percentage of explained inertia. Similarities in the fauna between particular lakes were also analysed by the single linkage method for the hierarchical clustering of objects. The distance measure is an Euclidean distance. The results are presented in the form of a dendrogram drawn for 40 lakes.

Relationships between the presence of beetles and the analysed environmental parameters in individual lakes were determined with the help of a principal component analysis, PCA. All calculations were performed in Statistica, ver. 13.0 (StatSoft, Tulsa, USA).

## Results

### General description of the collected material

**Figure 3:**
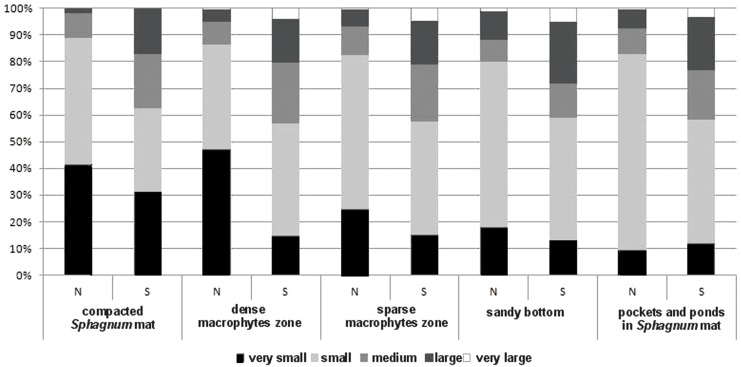
The structure of body size of Coleoptera in distinguished habitats. N, abundance; S, number of species.

The collected material comprised 10,139 specimens representing 124 species classified to seven families (Gyrinidae, Haliplidae, Noteridae, Dytiscidae, Hydraenidae, Hydrochidae and Hydrophilidae) (Appendix S1). Most species (55.6%) belonged to the two smallest body size classes. In total, they composed 84.75% of all collected specimens. The remaining beetles represented the consecutively higher body size classes, of which the largest one had the fewest representatives (Table 1). Most beetles (28.8% of the entire material) were collected in pockets and ponds of the *Sphagnum* mat. It was also there that the highest species richness was determined (91 spp.). The species diversity was lower in the dense macrophytes zone (26.6%; 74 spp.), sparse macrophytes zone (19.64%; 66 spp.), compacted *Sphagnum* mat (18.9%; 64 spp.), and finally in the sandy bottom (5.9%; 39 spp.). Very small beetles, which represented the first body size class, demonstrated the highest species richness in the compacted *Sphagnum* mat (41.5% of the whole collected material) (Fig. 3), while being the most numerous in the dense macrophyte zone. Small beetles (class 2 of the body size) were most numerously caught in the pockets and ponds within the *Sphagnum* mat (73.4%). The presence of very large beetles was notable in the sparse macrophytes zone, pockets and ponds in the *Sphagnum* mat and in the sandy bottom zone, although they made up a small share of the collected material (around 1%).

**Figure 4:**
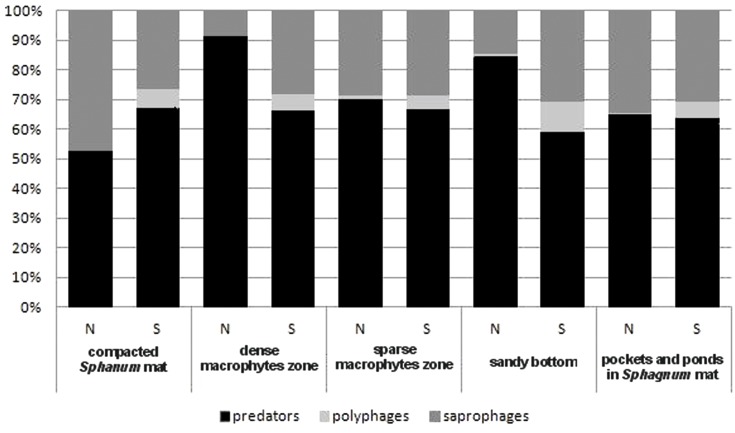
The trophic structure of Coleoptera in distinguished habitats. N, abundance; S, number of species.

In the trophic structure, the highest percentage (71.9%) was achieved by predatory beetles, represented mainly by Dytiscidae (Fig. 4). Although their species diversity was similar in all the habitats, they were most numerous in the dense macropytes zone (91.3%) and in the sandy bottom zone (84.5%). Most saprophages (47.2%) were noted in the compacted *Sphagnum* mat, while the number of species representing polyphages was low in all of the habitats, with the highest score (six spp.) determined in the compacted *Sphagnum* mat. The GLM Repeated Measure Anova results (*F*_(2,499)_ = 6.74, *p* = 0.0089) indicate significant differences in counts of the distinguished trophic groups in the particular habitats. The counts of predatory beetles in the habitats in question did not differ significantly (the Tukey’s HSD test, *p* = 0.18). Conversely, differences in the counts of predators and polyphages were significant (*p* = 0.0003).

### Dependencies between types of habitats, body size and abundance as well as biodiversity of Coleoptera

**Figure 5:**
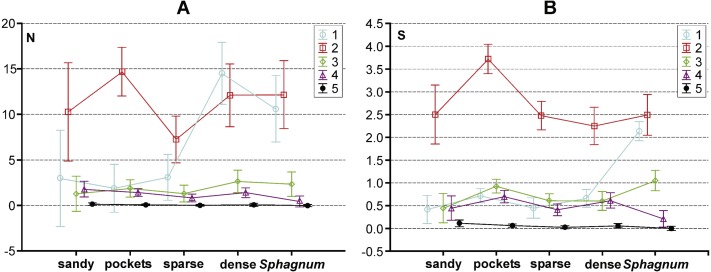
Results of a Tukey *post hoc* test for GLM repeated measure Anova. The diagram shows the influence of the statistically significant synergistic effect between habitats and body size classes on. (A) abundance and (B) number of species in the habitas distinguished with the analysed lakes. Habitats: sandy, sandy bottom zone; pockets, pockets and ponds in a *Sphagnum* mat; sparse, sparse macrophytes; dense, dense macrophytes; *Sphagnum*, compacted *Sphagnum* mat. (Bars indicate 0.95 confidence intervals). 1, very small beetles; 2, small beetles; 3, medium beetles; 4, large beetles; 5, very large beetles.

The results of the GLM repeated measure Anova analysis point to a significant influence of the synergistic effect between the type of habitat and body size class on the abundance of beetles (*F*_(16,1996)_ = 7.25, *p* = 0.0000002). Significant differences (the Tukey’s HSD *post*-*hoc* test) between the analysed subclasses are illustrated in Fig. 5A (cf. Appendix S3). Moreover, a significant influence of the synergistic effect was determined between the indicated factors (habitat and body size class) and the number of species (*F*_(16,1996)_ = 16.22, *p* = 0.00002) (Appendix S3). Results of the GLM repeated measure also point to a significant influence of the type of habitat on the number of beetles *F*_(4,499)_ = 6.75, *p* = 0.00003 (Fig. 5B). Significant differences (the Tukey’s HSD *post*-*hoc* test) were determined between the compacted *Sphagnum* mat and sparse macrophytes zone (*p* = 0.031), dense macrophytes zone and sparse macrophytes zone (*p* = 0.00014), dense macrophytes zone and pockets (*p* = 0.0479). Moreover, significant differences were demonstrated in the numbers of beetles representing the distinguished body size classes (*F*_(4,1996)_ = 77.61, *p* = 0.00002). The Tukey’s *post-hoc* test reveals significant differences in the counts of beetles representing all the classes of body size (*p* = 0.00002), except 3 and 4 class (*p* = 0.77). Both factors, the type of habitat (*F*_(4,499)_ = 11.04, *p* = 0.000003) and body size (*F*_(4,1996)_ = 375.61, *p* = 0.000001), also had a significant impact on the number of species determined. With respect to the species diversity, significant differences were found between the *Sphagnum* mat and sparse macrophytes (*p* = 0.0048), and between the pockets and ponds in the *Sphagnum* mat and sandy bottom zone (*p* = 0.0046), and between the *Sphagnum* mat and sparse macrophytes (*p* = 0.00018) and dense macrophytes (*p* = 0.0014). Significant differences in the number of species were shown between all body size classes (*p* = 0.00002), except 1 and 3 (*p* = 0.24).

Negative correlations were observed in all the habitats between the body size and the number of beetles. It was only in the sandy bottom zone that these correlations were not significant (*p* = 0.47) (Table 2). Similarly, negative correlations appeared between the body size and species richness in the compacted *Sphagnum* mat (*p* = 0.01) and in the sparse macrophytes zone (*p* = 0.0008) (Table 2).

**Table 2:** Results of the Pearson’s analysis of correlation (*r*_*p*_). Dependencies between body size and the abundance and diversity of Coleoptera in the distinguished habitats (Pearson’s analysis of correlation). Statistically significant data are in bold (*p* < 0.05).

Habitat	**Abundance**		**Number of species**	
	*r*_*p*_	*p*	*r*_*p*_	*p*
Compacted *Sphagnum* mat	−**0.140**	**0.011**	−**0.13**	**0.011**
Dense macrophytes zone	−**0.265**	**0.0008**	−0.04	0.40
Sparse macrophytes zone	−**0.265**	**0.00001**	−**0.18**	**0.0008**
Sandy bottom zone	–0.31	0.47	−0.09	0.48
Pockets and ponds in *Sphagnum* mat	−0.10	0.06	−0.04	0.40

### Dependencies between the type of habitats, body size and abundance of Coleoptera in lakes

**Figure 6:**
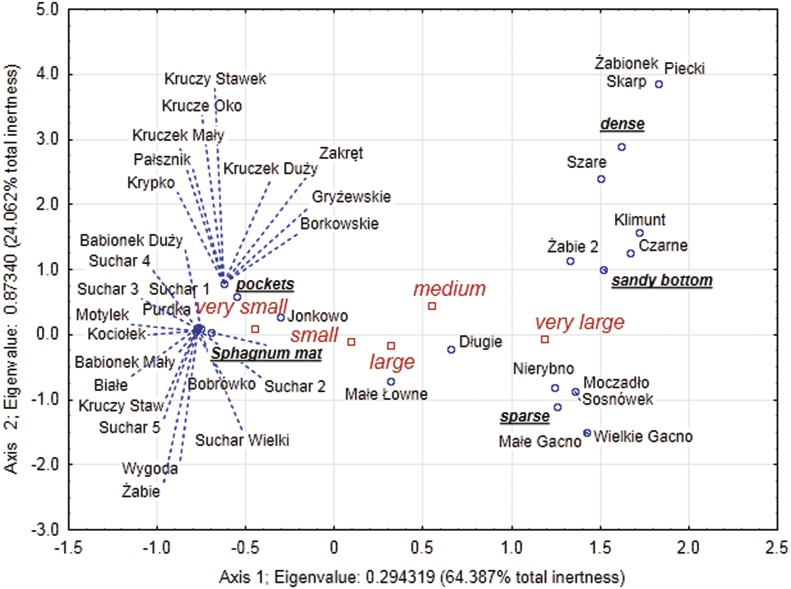
Multidimensional correspondence analysis (MCA). Relationship between identified classes of body size, distinguished habitats and individual lakes along the first and second MCA.

**Figure 7:**
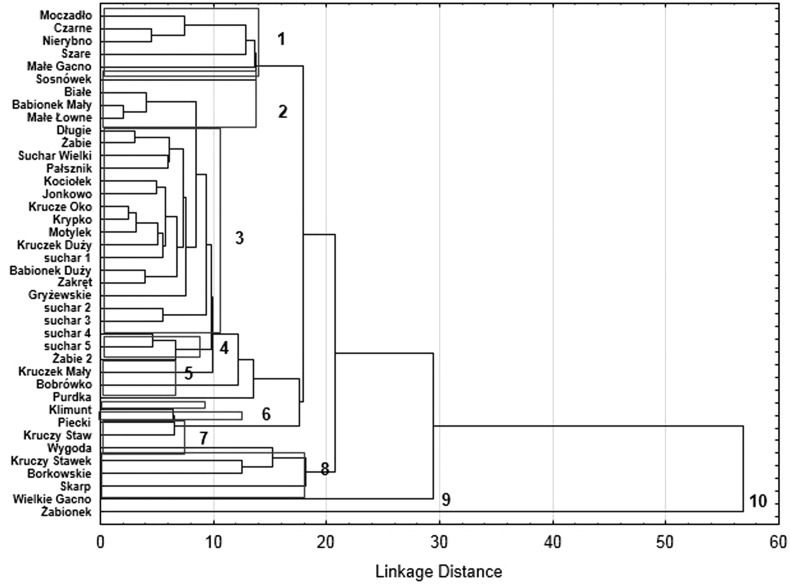
Results of dendrogram for 40 lakes. Single Linkage, Euclidean distances (1–10—cluster distinguished).

Our analysis of the body size structure of beetles in the fauna populating every lake demonstrated significant differences in the counts of beetles representing different body size classes (*x*^2^ = 171.18, *df* = 156; *p* = 0.0001). The plotted diagram points to lakes with a more strongly developed *Sphagnum* mat concentrating small organisms (two smallest body size classes) (Fig. 6). In the dendrogram (Fig. 7), these lakes create centrally located clusters (2–7). In the lakes less densely overgrown with *Sphagnum* sp., with the littoral zone either bare or weakly overgrown by sparse macrophytes, a larger contribution of organisms that belonged to the other body size classes, especially middle-size ones, was notable (clusters 8–9) (Figs. 6 and 7). Lakes included in cluster 1 are characterized by a minimal share of the smallest beetles (prevalent are the beetles that belong to the second body size class). The lakes Żabionek and Wielkie Gacno were distinguished by a demonstrably large contribution of big beetles.

### Presence of beetles in lakes versus the fractal structure

Our analysis of the correlations confirmed a strong positive correlation between the development of a *Sphagnum* mat in the littoral zone of each lake and the abundance of beetles which belong to the first body size class (*r*_*p*_ = 0.7025, *p* = 0.000013), while the correlation with the number of the largest beetles was negative (*r*_*p*_ =  − 0.21, *p* = 0.04). Both releationships were linear: LOG (Body size 1) = 0.9045 + 0.4736 * LOG (*Sphagnum* mat%); LOG (Body size 5) = 0.1531–0.101 * LOG (*Sphagnum* mat%). The remaining correlations were not significant statistically (Table 3).

**Table 3:** Results of the Pearson’s analysis of correlation. Changes in the abundance (LOG N) of the distinguished body size and the growth of the *Sphagnum* mat (LOG %) in lakes. Statistically significant data are in bold (*p* < 0.05).

**Variables**	*r*_*p*_	*r*^2^	*t*	*p*	
Body size class 1 & *Sphagnum* mat (%)	**0.702**	**0.449**	**6.085**	**0.00004**	
Body size class 2 & *Sphagnum* mat (%)	−0.19	0.03	−1.236	0.22	
Body size class 3 & *Sphagnum* mat (%)	−0.155	0.02	−0.97	0.338	
Body size class 4 & *Sphagnum* mat (%)	−0.16	0.027	−1.03	0.308	
Body size class 5 & *Sphagnum* mat (%)	−**0.207**	**0.043**	−**1.307**	**0.04**	

**Figure 8:**
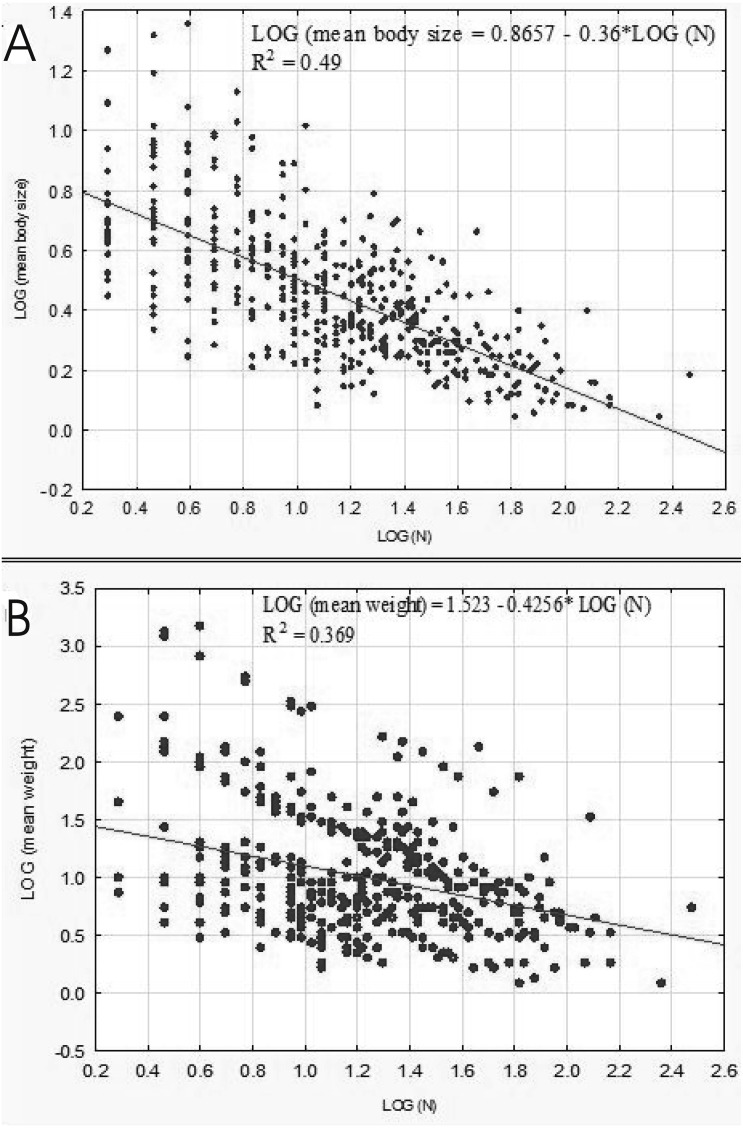
Linear regression for Coleoptera in lakes. Abundance vs. body size (A) and abundance vs. weight (B).

A negative relationship between the size of body and the number of organisms in lakes emerged from our analysis conducted on the level of samples (*r*_*p*_ = 0.7046, *p* = 0.00001). This correlation was linear as well (Fig. 8A). The relationship between the weight and abundance of beetles was similar in character (Fig. 8B).

**Figure 9:**
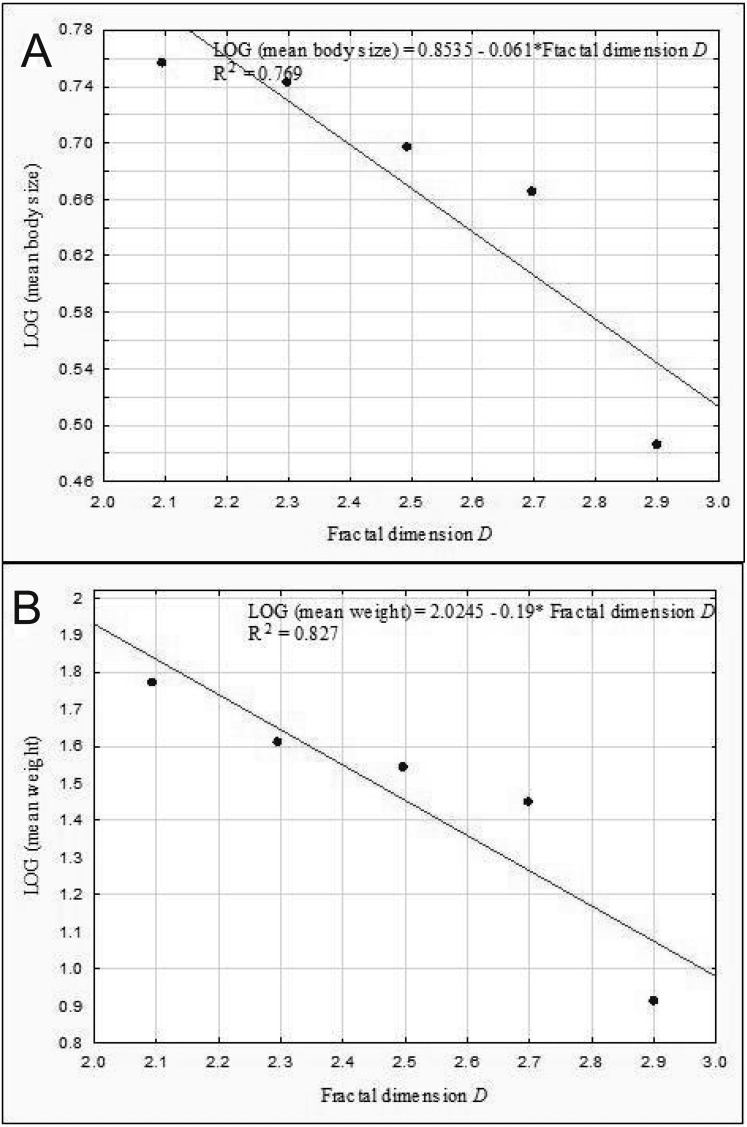
Linear regression for Coleoptera in lakes. Mean body size vs fractal dimension (A) and mean weight vs. fractal dimension (B).

The results of the linear regression analysis showed that as the fractal dimension increased, the mean body size (*r*_*P*_ =  − 0.86, *p* = 0.037) and the mean weight of Coleoptera (*r*_*P*_ =  − 0.91, *p* = 0.032) decreased (Fig. 9). In turn, the relationship between the total biomass of beetles and the fractal dimension was not significant (*r*_*P*_ =  − 0.31, *p* = 0.623).

**Figure 10:**
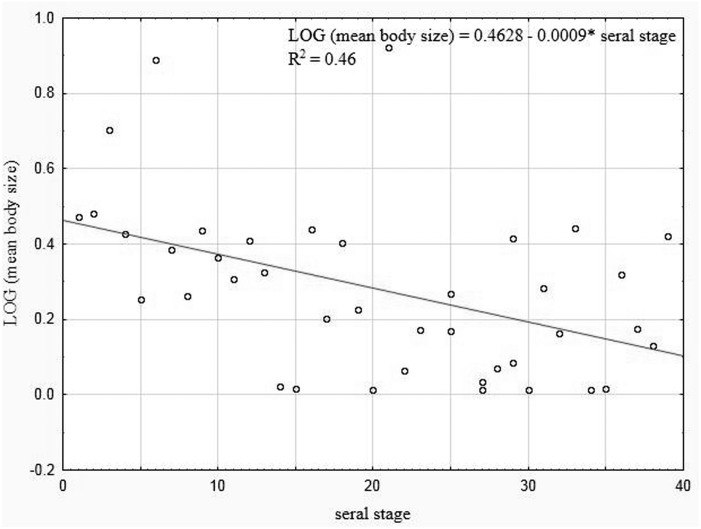
Linear regression for Coleoptera in lakes. Mean body size vs. seral stage of successsion.

Our analysis performed for all the lakes (arranged according to the seral stages of succession), which took into account the extent of the *Sphagnum* mat cover and the shares of the other habitats with different fractal dimensions, also indicated negative correlations between the seral stage of succession and the mean body size (*r*_*P*_ =  − 0.465, *p* = 0.0034) (Fig. 10).

**Figure 11:**
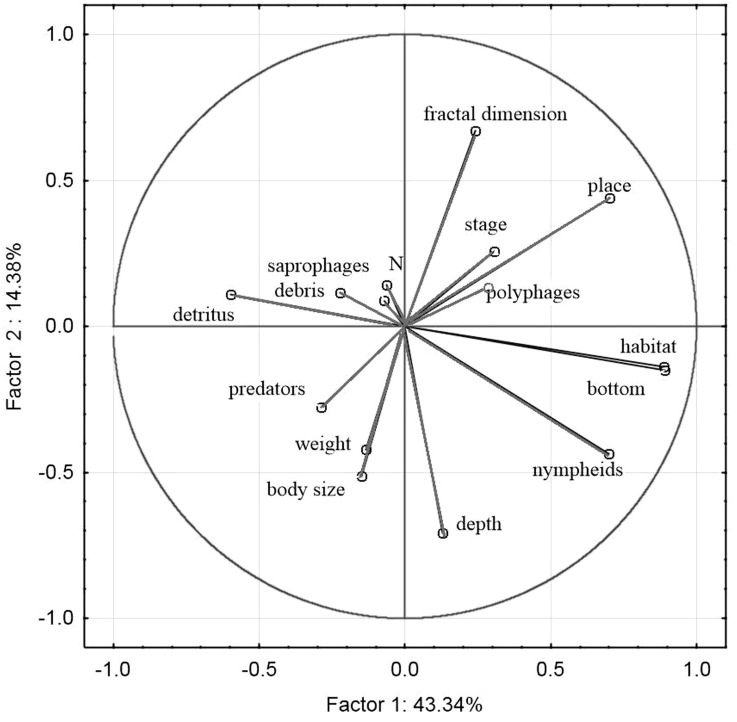
Principal Component Analysis (PCA) ordination plot of functional groups, and environmental variables in samples along the first and second PCA axis

The principal component analysis of the variables representing the parameters of habitats and trophic groups distinguished in our study (Fig. 11) suggests that the first axis corresponding to the highest own values most strongly corresponds with the variables ‘predators’ and ‘saprophages’, while the second axis shows the strongest correlations with the variables ‘body size’, ‘weight’, ‘N—abundance’, ‘place’, ‘depth’, ‘stage’ and ‘fractal dimension’. Positive correlations were determined between ‘abundance’ and fractal dimension (*r*_*p*_ = 0.26, *p* = 0.003), ‘abundance’ and ‘stage’ (*r*_*p*_ = 0.06, *p* = 0.001), ‘saprophages’ and ‘detritus’ (*r*_*p*_ = 0.75, *p* = 0.004), ‘polyphages’ and ‘fractal dimension’ (*r*_*p*_ = 0.81, *p* = 0.003), while negative correlations appeared between ‘abundance’ and ‘depth’ (*r*_*p*_ =  − 0.41, *p* = 0.003), ‘saprophages’ and ‘depth’ (*r*_*p*_ =  − 0.85, *p* = 0.001), ‘abundance’ and ‘weight’ and ‘body size’ (*r*_*p*_ =  − 0.15, *p* = 0.008) and between ‘predators” and place (*r*_*p*_ =  − 0.85, *p* = 0.004) and between ‘abundance’ and ‘nympheids’ (*r*_*p*_ =  − 0.78, *p* = 0.006).

## Disscussion

### Influence of the habitat and architecture on the richness, abundance and body size of Coleoptera

This article is an outcome of 12 years of field studies, during which time we acquired a very rich amount of comparative material (124 species) from 40 lakes. Among the numerous references dedicated to water beetles, there are few which document a larger number of lakes (e.g., Nilsson & Söderberg, 1996; Buczyński, 2012).

In our study, same as Linzmeier & Ribeiro-Costa (2011), we observed the highest species diversity and density of beetles representing two smallest body size classes, mostly of the genus *Hydroporus*. According to Ulrich (2007), empirical studies show the highest species richness among the medium-size species, while simulation models point to the smallest species. A contrary opinon is held by Scheffer & Van Nes (2006) and Scheffer et al. (2015), who maintain that intermediate body sizes in animal assemblages are found extremely rarely because coevolution in competitive systems favours the co-occurence of species either very similar to or very different from one another.

Thus, it seems to be the matter of assigning the criteria distinguishing body size categories. At the same time, we demonstrated very scarce presence of the largest species, a finding also reported by Nilsson & Söderberg (1996), Ulrich (2007) and Linzmeier & Ribeiro-Costa (2011).

Similarly to Nilsson & Söderberg (1996), Saint-Germain et al. (2007) and Linzmeier & Ribeiro-Costa (2011), we revealed negative correlations between the body size (and the individual body mass) and the abundance of beetles, as well as between the body size and the species richness. According to Nilsson & Söderberg (1996), a negative relationship between the abundance and body size is typical for most aquatic organisms. On the other hand, Saint-Germain et al. (2007) emphasise the negative correlation between the abundance of species and body size.

For clarification, we made an in-depth analysis of the dependencies between the body size and the number of beetles relative to the aspects defining the complex nature of each habitat. Such an approach enjoys a long tradition, as suggested by Ulrich (2007), although it is rarely implemented in hydrobiological research (Cremona, Planas & Lucotte, 2008; Tokeshi & Arakaki, 2012; Désamoré et al., 2018). We demonstrated significant differences between the abundance and species richness of beetles representing different body size classes in the particular habitats. Figure 5 shows that the highest abundance and species richness occurred in the *Sphagnum* mat and mostly with respect to the smallest beetles (body size classes 1 and 2), mainly of the genera *Anacaena* and *Hydroporus*. In the same habitat, the least numerous were large beetles and the largest ones, such as *Dytiscus* or *Cybister*, were completely absent. A reverse situation was observed in the sandy bottom zone and in the pockets and ponds within the *Sphagnum* mat, where the smallest beetles (class 1) were the least numerous, while the largest ones were more abundant than elsewhere. Medium-size beetles (classes 2 and 3), e.g., *Noterus*, *Agabus*, and *Ilybius*, were numerous in all habitats. These relationships are explained perfectly well by the fractal structure. Should we take into consideration an increase in the fractal dimension from the smallest one (the less complex form of a habitat) in the sandy bottom zone to the highest (the more complex form of a habitat with increasingly small structural elements) in the *Sphagnum* mat, then our observations are in accord with the ones reported by other hydrobiologists, who conclude that the species diversity and density increase as the fractal dimensions increases (e.g., Verberk et al., 2001; Tokeshi & Arakaki, 2012). Meanwhile, small spaces between leaves in a *Sphagnum* mat inhibit the presence of very large beetles, a conclusion supported by the results provided by Tokeshi & Arakaki (2012). Similar conclusions were drawn by Scheffer et al. (2015), who suggest that the maximum body size is limited by the available space in which beetles could move.

In our study, negative correlations between the body size, weight or abundance of water beetles were determined in particular habitats. Considering the total biomass of beetles in individual habitats, we were unable to identify any significant differences, which again agrees with the conclusions drawn by Tokeshi & Arakaki (2012), namely that biomass does not change in the fractal dimensions. An increase in the density of beetles is offset by a decrease in the individual size of the body, which is concordant with the results of Tokeshi & Arakaki’s research (2012).

A very compact structure of vegetation creates niches for small beetles, offering them egg-laying safety, food for larvae (much humus) and a shelter from predators (Verberk et al., 2001). How is the co-occurrence of these smallest beetles possible if, according to Scheffer et al. (2015), specimens of the same size compete with one another most strongly? Some hydrobiologists, e.g., Scheffer & Van Nes (2006) and Scheffer et al. (2015), draw attention to the fact that these organisms create self-organising assemblages, the presence of which is the result of co-evolution between potential competitors.

In the trophic structure we analysed we discovered that 70% of beetles were predators, being the most numerous groups in all habitats. According to Bloechl et al. (2010), the number of predatory beetles depends on the amount of their prey. In our study, the smallest quantitative impact of predatory beetles (same as the number of saprophages) was identified in the *Sphagnum* mat. There they represented classes of organisms with small body size, e.g., *Hydroporus*, which do not limit the presence of other water beetles, as they use other food resources, e.g., zooplankton, smaller insects, like mayflies, or insect eggs (Frelik & Pakulnicka, 2015; Frelik, Koszałka & Pakulnicka, 2016; Perissinotto, Bird & Bilton, 2016). According to Scheffer & Van Nes (2006), Pakulnicka et al. (2013) and Scheffer et al. (2015), what happens here is the evident division of functions, which relies on the principle of minimising similarity (being similar *albeit* different), as this minimizes competitiveness. As Scheffer et al. (2015) maintain, these mechanisms ensure a certain measure of redundancy of similar species in the environment, which is essential for the functioning of ecosystems during unfavourable changes.

### Implications of the disharmonic succession of lakes on coleopteran fauna

The species diversity and density of organisms are the properties most often applied to measuring both biodiversity and quality of the environment, also in the context of succession changes (Kowalik, 1968; Ranta, 1985; Kordylas, 1990; Linzmeier & Ribeiro-Costa, 2011). According to Vamosi, Naydani & Vamosi (2007), species richness and body size change predictably along the spatial gradient, whereas Linzmeier & Ribeiro-Costa (2011) suggest that changes in the body size can be an important indicator, while Saint-Germain et al. (2007) claim that body mass is a better measure than abundance.

Lake succession is a phenomenon that occurs in a given place and over a certain period of time, and with time (in subsequent stages) habitat conditions, mostly shaped by macrophytes, change as well. For particular organisms, optimal living conditions appear here and now, and then they disappear. Thus, changes in the primary structure of lakes result in changes in the secondary structure, formed by assemblages of various organisms. Many ecologists, including Siemann, Haarstad & Tilman (1999), Brown (2003), White et al. (2007) and Linzmeier & Ribeiro-Costa (2011), underline that body size is correlated with numerous morphological, physiological and ecological features, such as an ability to disperse, metabolic capacity, digestion capacity, reproduction rate and duration of a generation, as well as biodiversity. Scheffer & Van Nes (2006) claim that these characteristics act as specific mechanisms which prevent competition between species, especially ones with similar body size.

We have demonstrated that body size and body mass are also useful measurements in investigations into the mechanisms of succession of beetles in dystrophic lakes. Communities of lake-dwelling beetles representing different seral stages are characterised by diverse shares of species representing different body size classes. This is a consequence of changes in the fractal structure, that is the representativeness of habitats (fractals) with different fractal dimensions. Hence, in young lakes (see the Dendrogram), where the littoral zone is very modestly overgrown with plants, we noted very few smallest beetles associated with peats (tyrphophiles), e.g., *Anacaena*, *Helophorus* or *Hydroporus,* which agrees with the results reported by Bloechl et al. (2010)*.* However, these beetles were very numerous in mature lakes, where a *Sphagnum* mat dominates. Thus, we determined negative correlations between the body size and the percentage of an area overgrown with *Sphagnum* moss in the littoral zone of lakes, as well as between the body size (and mean weight) and the abundance of beetles in lakes. Also, we observed that as the fractal dimension of lakes increased, the mean body size (and mean weight) decreased. As spaces between the habitat’s components, i.e., plants, decrease in size and become more and more complex, smaller organisms are evidently preferred, even though larger ones may occur in such habitats as well, because they can push away or move plant stems, small shoots or leaves, an observation also made by McAbendroth et al. (2005). This could be the reason why large beetles were found in the pockets and ponds within the *Sphagnum* mat, and in the ecotone between the *Sphagnum* mat and the open water surface.

We support the view of other researchers, e.g., McAbendroth et al. (2005), Bloechl et al. (2010), Linzmeier & Ribeiro-Costa (2011), and believe that large species gain benefits during the early stages of succession, while smaller and specialised ones are at an advantage in the final stages. We have shown (see Fig. 11) that the abundance of beetles is correlated with the age of lakes (the seral stage) (contrary to Bloechl et al. (2010), and with the fractal dimension (McAbendroth et al., 2005). These relationships are connected with the development of plants, which offer a shelter from predators, places for laying eggs and food supply. Like Bloechl et al. (2010) and Cremona, Planas & Lucotte (2008), we demonstrated a relationship between polyphages (Haliplidae) and submerged, more compact plants, while a negative correlation was proven with nympheids, which restrain the growth of food (algae) eaten by polyphages. In turn, the presence of saprophages is correlated with detritus and drifters (which additionally contribute to a growth in the fractal dimension by creating microhabitats that can be used by smaller organisms), which has been confirmed by Verberk et al. (2001) and Cremona, Planas & Lucotte (2008). Moreover, we found out that the abundant presence of predatory beetles in all lakes, and especially among loose vegetation, is favoured by the availability of potential prey, a conclusion also supported by the findings made by Bloechl et al. (2010), whereas a more compact structure is clearly a barrier to the occurrence of the largest organisms. Another factor that limits the number of beetles is the depth. This suggestion is confirmed by the research completed by Brittain (1978), Pakulnicka, Górski & Bielecki (2015b), Pakulnicka et al. (2013) and Pakulnicka et al. (2015a). Among the lakes which we analysed, the ones that contained dark brown water with acidic reaction (Pakulnicka & Zawal, 2018) there were no fish or amphibians, which are usually considered as one of the major factors limiting the presence of water beetles (Eriksson et al., 1980; Solé & Rödder, 2010).

## Conclusions

Our research has revealed that an application of fractal concepts enables a more comprehensive explanation regarding the character of fauna relative to the complex natural aspects of habitats. This approach is also helpful in explaining directional changes in fauna induced by the natural succession of lakes. The taxonomic diversity and various densities of beetles in lakes increase as the fractal dimension increases. There are negative correlations between the body size and abundance. An increase in the density of beetles in fractals with a higher dimension is offset by a lower individual body size of specimens. Consequently, the biomass is constant regardless of the fractal dimension.

##  Supplemental Information

10.7717/peerj.5662/supp-1 Appendix S1 General characteristics of the lakes %—contribution in the littoral zone (Sm, *Sphagnum* mat; Di, Sparse macrophytes; De, Dense macrophytes; A, sandy bottom); T, Stage of succession (O, oligohumic, M, mesohumic; P, polyhumic), S, number of species.

10.7717/peerj.5662/supp-2 Appendix S2 Quantitative occurrence of beetles in humic lake N,number of individuals, NS, number of samples; L,number of lakes; Bs, body size class; F, functional group (P, predators; S, saprophages, F, polyphages); P, Legal protection, EN,VU; LC, threat status (Polish Red List); min, minimum abundance of the species in a single sample (excluded samples where the species did not occur); max, abundance of the species in a single sample; Mean, average abundance of the species in a single sample; SD, standard deviation.

10.7717/peerj.5662/supp-3 Appendix S3 Results of HSD Tuckey *post hoc* test for GLM repeated measure Anova Influence of significant interaction between habitats and Body size on (A) abundance of beetles, (B) number of species. SN, Subclass number; BS, Body size class (1-very small beetles, 2-small, 3-medium, 4-large, 5-very large); Habitat: Sphagnum, compacted Sphagnum mat; dense, dense macrophytes zone; sparse, sparse macrophytes zone,

10.7717/peerj.5662/supp-4 Appendix S4 Database - source data

## Additional Information and Declarations

### Field Study Permissions

The following information was supplied relating to field study approvals (i.e., approving body and any reference numbers): Field studies were conducted in accordance with field study approvals: Certificate of RDOS in Olsztyn (WOPN.070.21.2018.AKI), WNP certificate (PNE 08-070 / 18) and ZPK Certificate (060.1.2018).

### Data Availability

The following information was supplied regarding data availability: The raw data are provided in Appendix S4.
