# Supplementary material for: Effect of changes in the fractal structure of a littoral zone in the course of lake succession on the abundance, body size sequence and biomass of beetles
Source: PeerJ. 2018 Sep 26;6:e5662. doi: 10.7717/peerj.5662 (PMC6163033; doi:10.7717/peerj.5662)
Supplement: Appendix S2 — N,number of individuals, NS, number of samples; L,number of lakes; Bs, body size class; F, functional group (P, predators; S, saprophages, F, polyphages); P, Legal protection, EN,VU; LC, threat status (Polish Red List); min, minimum abundance of the species in a single sample (excluded samples where the species did not occur); max, abundance of the species in a single sample; Mean, average abundance of the species in a single sample; SD, standard deviation. [file peerj-06-5662-s002.docx]

**Appendix 2 Quantitative occurrence of beetles in humic lake**. N – number of individuals, NS – number of samples. L – number of lakes, Bs – body size class, F – functional group (P – predators, S – saprophages, F – polyphages), P – Legal protection, EN,VU, LC – threat status (Polish Red List), min – minimum abundance of the species in a single sample (excluded samples where the species did not occur), max – abundance of the species in a single sample, Mean – average abundance of the species in a single sample, SD – standard deviation.

| **Species** | **N** | **NS** | **Mean ± SD** | **min** | **max** | **L** | **Bs** | **F** |
| --- | --- | --- | --- | --- | --- | --- | --- | --- |
| *Gyrinus aeratus Steph.* | 142 | 11 | 2.9 ± 33.5 | 1 | 114 | 5 | 3 | P |
| *Gyrinus marinus Gyll.* | 23 | 6 | 3.8 ± 2.9 | 1 | 9 | 2 | 3 | P |
| *Gyrinus natator (L.)* | 1 | 1 | 1.0 | 1 | 1 | 1 | 3 | P |
| *Gyrinus paykulli Ochs* | 2 | 2 | 1.0 | 1 | 1 | 2 | 3 | P |
| *Gyrinus substriatus Steph.* | 7 | 6 | 1.2 ± 0.4 | 1 | 2 | 6 | 3 | P |
| *Gyrinus suffriani Scriba* | 5 | 3 | 1.7 ± 1.2 | 1 | 3 | 3 | 3 | P |
| *Haliplus confinis Steph.* | 2 | 2 | 1.0 ± 0.0 | 1 | 1 | 2 | 2 | F |
| *Haliplus flavicollis Sturm* | 18 | 13 | 1.4 ± 1.4 | 1 | 6 | 5 | 2 | F |
| *Haliplus fluviatilis Aubé* | 4 | 3 | 1.3 ± 0.6 | 1 | 2 | 2 | 2 | F |
| *^VU^Haliplus fulvicollis Er.* | 5 | 3 | 1.7 ± 1.2 | 1 | 3 | 2 | 2 | F |
| *Haliplus immaculatus Gerh.* | 2 | 2 | 1.0 ± 0.0 | 1 | 1 | 2 | 2 | F |
| *Haliplus ruficollis (De G.)* | 12 | 11 | 1.1 ± 0.3 | 1 | 2 | 9 | 2 | F |
| *Haliplus wehnckei Gerh.* | 1 | 1 | 1.0 | 1 | 1 | 1 | 2 | F |
| *Peltodytes caesus (Duft.)* | 1 | 1 | 1.0 | 1 | 1 | 1 | 2 | F |
| *Noterus clavicornis (De G.)* | 7 | 5 | 1.4 ± 0.9 | 1 | 3 | 5 | 2 | P |
| *Noterus crassicornis (O.F. Müll.)* | 2532 | 312 | 8.1 ± 12.3 | 1 | 131 | 34 | 2 | P |
| *Agabus affinis (Payk.)* | 37 | 10 | 3.7 ± 3.2 | 1 | 9 | 3 | 3 | P |
| *Agabus congener (Thunb.)* | 3 | 3 | 1.0 ± 0.0 | 1 | 1 | 3 | 3 | P |
| *Agabus fuscipennis (Payk.)* | 5 | 1 | 5.0 | 5 | 5 | 1 | 3 | P |
| *Agabus labiatus (Brahm)* | 1 | 1 | 1.0 | 1 | 1 | 1 | 3 | P |
| *Agabus undulatus (Schrank)* | 10 | 6 | 1.7 ± 1.0 | 1 | 3 | 5 | 3 | P |
| *Platambus maculatus (L.)* | 1 | 1 | 1.0 | 1 | 1 | 1 | 3 | P |
| *Ilybius aenescens Thoms.* | 4 | 4 | 1.0 ± 0.0 | 1 | 1 | 3 | 3 | P |
| *Ilybius ater (De G.)* | 16 | 12 | 1.3 ± 0.7 | 1 | 3 | 7 | 4 | P |
| *Ilybius fenestratus (Fabr.)* | 273 | 99 | 2.8 ± 3.1 | 1 | 19 | 17 | 4 | P |
| *Ilybius fuliginosus (Fabr.)* | 3 | 3 | 1.0 ± 0.0 | 1 | 1 | 3 | 4 | P |
| *Ilybius guttiger (Gyll.)* | 5 | 5 | 1.0 ± 0.0 | 1 | 1 | 2 | 3 | P |
| *Ilybius obscurus (Marsh.)* | 2 | 1 | 2.0 | 2 | 2 | 1 | 4 | P |
| *Ilybius quadriguttatus (Lacord.)* | 6 | 5 | 1.2 ± 0.4 | 1 | 2 | 3 | 4 | P |
| *Ilybius similis Thoms.* | 3 | 1 | 3.0 | 3 | 3 | 1 | 4 | P |
| *Ilybius subaeneus Er.* | 8 | 8 | 1.0 ± 0.0 | 1 | 1 | 6 | 4 | P |
| *Colymbetes fuscus (L.)* | 4 | 4 | 1.0 ± 0.0 | 1 | 1 | 3 | 4 | P |
| *Colymbetes paykulli Er.* | 15 | 3 | 5.0 ± 3.6 | 2 | 9 | 2 | 4 | P |
| *Colymbetes striatus (L.)* | 9 | 2 | 4.5 ± 2.1 | 3 | 6 | 1 | 4 | P |
| *Rhantus bistriatus (Bergst.* | 1 | 1 | 1.0 | 1 | 1 | 1 | 4 | P |
| *Rhantus exsoletus (Forst.)* | 1 | 1 | 1.0 | 1 | 1 | 1 | 4 | P |
| *Rhantus grapii (Gyll.)* | 3 | 3 | 1.0 ± 0.0 | 1 | 1 | 3 | 4 | P |
| *Rhantus latitans Sharp* | 10 | 5 | 2.0 ± 1.2 | 1 | 4 | 4 | 4 | P |
| *Rhantus notaticollis (Aubé)* | 1 | 1 | 1.0 | 1 | 1 | 1 | 4 | P |
| *Rhantus notatus (Fabr.)* | 8 | 4 | 2.0 ± 1.7 | 1 | 4 | 4 | 4 | P |
| *R. suturalis (Mac L.)* | 12 | 9 | 1.3 ± 0.7 | 1 | 3 | 9 | 4 | P |
| *Liopterus ruficollis (Fabr.)* | 1 | 1 | 1.0 | 1 | 1 | 1 | 3 | P |
| *Acilius canaliculatus (Nic.)* | 57 | 27 | 2.1 ± 1.5 | 1 | 7 | 13 | 4 | P |
| *Acilius sulcatus (L.)* | 70 | 25 | 2.8 ± 4.0 | 1 | 17 | 12 | 4 | P |
| *^P^Graphoderus bilineatus (De G.)* | 4 | 3 | 1.3 ± 0.6 | 1 | 2 | 2 | 4 | P |
| *Graphoderus cinereus (L.)* | 29 | 18 | 1.6 ± 1.2 | 1 | 5 | 10 | 4 | P |
| *Graphoderus zonatus (Hoppe)* | 7 | 5 | 1.4 ± 0.5 | 1 | 2 | 4 | 4 | P |
| *Cybister lateralimarginalis (De G.)* | 11 | 3 | 3.7 ± 2.5 | 1 | 6 | 1 | 5 | P |
| *Dytiscus circumcinctus Ahr.* | 1 | 1 | 1.0 | 1 | 1 | 1 | 5 | P |
| *Dytiscus dimidiatus Bergst.* | 4 | 4 | 1.0 ± 0.0 | 1 | 1 | 2 | 5 | P |
| *^P. VU^ Dytiscus lapponicus Gyll.* | 4 | 2 | 2.0 ± 1.4 | 1 | 3 | 2 | 5 | P |
| *Dytiscus marginalis ( L. )* | 16 | 12 | 1.3 ± 0.8 | 0 | 3 | 7 | 5 | P |
| *Hydaticus aruspex Clark* | 2 | 2 | 1.0 ± 0.0 | 1 | 1 | 2 | 4 | P |
| *Hydaticus modestus Sharp* | 1 | 1 | 1.0 | 1 | 1 | 1 | 4 | P |
| *Hydaticus seminiger (De G.)* | 16 | 10 | 3.1 ± 0.7 | 1 | 3 | 8 | 4 | P |
| *Nebrioporus canaliculatus (Lacord.)* | 1 | 1 | 1.0 | 1 | 1 | 1 | 2 | P |
| *Graptodytes bilineatus (Sturm)* | 1 | 1 | 1.0 | 1 | 1 | 1 | 1 | P |
| *Graptodytes pictus (Fabr.)* | 1822 | 93 | 19.6 ± 33.2 | 1 | 220 | 16 | 1 | P |
| *Hydroporus angustatus Sturm* | 55 | 39 | 1.4 ± 0.8 | 1 | 4 | 20 | 2 | P |
| *Hydroporus dorsalis (Fabr.)* | 8 | 8 | 1.0 ± 0.0 | 1 | 1 | 5 | 3 | P |
| *^VU^Hydroporus elongatulus Sturm* | 1 | 1 | 1.0 | 1 | 1 | 1 | 2 | P |
| *Hydroporus erythrocephalus (L.)* | 45 | 36 | 1.3 ± 0.8 | 1 | 5 | 14 | 2 | P |
| *Hydroporus gyllenhalii Sch.* | 2 | 1 | 2.0 | 2 | 2 | 1 | 3 | P |
| *Hydroporus incognitus Sharp* | 37 | 16 | 2.3 ± 2.2 | 1 | 8 | 11 | 2 | P |
| *^EN^Hydroporus melanocephalus (Marsh.)* | 1 | 1 | 1.0 | 1 | 1 | 1 | 2 | P |
| *Hydroporus memnonius Nic.* | 5 | 4 | 1.3 ± 0.5 | 1 | 2 | 2 | 2 | P |
| *Hydroporus neglectus Schaum* | 97 | 41 | 2.4 ± 4.1 | 1 | 26 | 16 | 1 | P |
| *Hydroporus notatus Sturm* | 2 | 2 | 1.0 ± 0.0 | 1 | 1 | 2 | 2 | P |
| *Hydroporus obscurus Sturm* | 257 | 90 | 2.9 ± 2.7 | 1 | 14 | 16 | 2 | P |
| *Hydroporus palustris (L.)* | 75 | 12 | 6.3 ±15.8 | 1 | 56 | 10 | 2 | P |
| *Hydroporus pubescens (Gyll.)* | 1 | 1 | 1.0 | 1 | 1 | 1 | 2 | P |
| *Hydroporus rufifrons (Duft.)* | 1 | 1 | 1.0 | 1 | 1 | 1 | 3 | P |
| *Hydroporus scalesianus Steph.* | 23 | 18 | 1.3 ± 0.6 | 1 | 3 | 13 | 1 | P |
| *Hydroporus striola (Gyll.)* | 2 | 2 | 1.0 ± 0.0 | 1 | 1 | 2 | 2 | P |
| *Hydroporus tristis Payk.* | 377 | 94 | 4.0 ± 6.8 | 1 | 52 | 24 | 2 | P |
| *Hydroporus umbrosus (Gyll.)* | 38 | 24 | 1.6 ± 1.3 | 1 | 7 | 9 | 1 | P |
| *Porhydrus lineatus (Fabr.)* | 18 | 14 | 1.3 ± 0.6 | 1 | 3 | 12 | 2 | P |
| *Hygrotus decoratus (Gyll.)* | 33 | 21 | 1.6 ± 1.6 | 1 | 8 | 11 | 1 | P |
| *Hygrotus impressopunctatus (Schall.)* | 21 | 18 | 1.2 ± 0.4 | 1 | 2 | 14 | 3 | P |
| *Hygrotus inaequalis (Schall.)* | 65 | 44 | 1.5 ± 0.9 | 1 | 5 | 20 | 2 | P |
| *Hygrotus versicolor (Schall.)* | 226 | 50 | 4.5 ± 5.3 | 1 | 27 | 4 | 2 | P |
| *Hyphydrus ovatus (L.)* | 319 | 119 | 2.7 ± 3.3 | 1 | 26 | 23 | 2 | P |
| *Hydroglyphus geminus (Fabr.)* | 76 | 22 | 3.5 ± 6.7 | 1 | 30 | 9 | 1 | P |
| *Bidessus hamulatus (Gyll.)* | 1 | 1 | 1.0 | 1 | 1 | 1 | 1 | P |
| *Bidessus unistriatus Goeze* | 6 | 5 | 1.2 ± 0.4 | 1 | 2 | 3 | 1 | P |
| *Laccophilus hyalinus ( Degeer )* | 3 | 3 | 1.0 ± 0.0 | 1 | 1 | 1 | 2 | P |
| *Laccophilus minutus (L.)* | 60 | 34 | 1.8 ± 1.3 | 1 | 6 | 16 | 2 | P |
| *Laccophilus poecilus (Klug)* | 51 | 3 | 17.0 ± 11.0 | 6 | 28 | 1 | 2 | P |
| *Helophorus fulgidicollis Motsch.* | 2 | 1 | 2.0 | 2 | 2 | 1 | 2 | S |
| *Helophorus granularis (L.)* | 6 | 6 | 1.0 ± 0.0 | 1 | 1 | 5 | 2 | S |
| *Helophorus griseus Herbst* | 1 | 1 | 1.0 | 1 | 1 | 1 | 2 | S |
| *Helophorus minutus Fabr.* | 38 | 25 | 1.5 ± 1.1 | 1 | 6 | 13 | 2 | S |
| *Helophorus pumilio Er.* | 2 | 2 | 1.0 ± 0.0 | 1 | 1 | 2 | 2 | S |
| *Hydrochus angustatus Germ.* | 2 | 1 | 2.0 | 2 | 2 | 1 | 2 | S |
| *Hydrochus brevis (Herbst)* | 4 | 4 | 1.0 ± 0.0 | 1 | 1 | 4 | 2 | S |
| *Hydrochus crenatus (Fabr.)* | 39 | 25 | 1.6 ± 1.4 | 1 | 8 | 8 | 2 | S |
| *Hydrochus elongatus (Schall.)* | 5 | 5 | 1.0 ± 0.0 | 1 | 1 | 3 | 2 | S |
| *Hydrochus ignicollis Motsch.* | 1 | 1 | 1.0 | 1 | 1 | 1 | 2 | S |
| *Hydrochus nitidicollis Muls.* | 8 | 5 | 1.6 ± 1.3 | 1 | 4 | 3 | 2 | S |
| *Anacaena limbata (Fabr.)* | 27 | 9 | 3.0 ± 2.9 | 1 | 10 | 5 | 1 | S |
| *Anacaena lutescens (Steph.)* | 1192 | 226 | 5.3 ± 8.9 | 1 | 63 | 35 | 1 | S |
| *Berosus signaticollis (Charp.)* | 1 | 1 | 1.0 | 1 | 1 | 1 | 3 | S |
| *Cymbiodyta marginella (Fabr.)* | 4 | 2 | 2.0 ± 1.4 | 1 | 3 | 1 | 3 | S |
| *Enochrus affinis (Thunb.)* | 289 | 99 | 2.9 ± 3.1 | 1 | 18 | 23 | 2 | S |
| *Enochrus coarctatus (Gredl.)* | 284 | 115 | 2.5 ± 2.8 | 1 | 18 | 24 | 2 | S |
| *Enochrus nigritus (Sharp)* | 1 | 1 | 1.0 | 1 | 1 | 1 | 2 | S |
| *Enochrus melanocephalus (Ol.)* | 1 | 1 | 1.0 | 1 | 1 | 1 | 3 | S |
| *Enochrus ochropterus (Marsh.)* | 145 | 72 | 2.0 ± 1.7 | 1 | 8 | 23 | 3 | S |
| *Enochrus quadripunctatus (Herbst)* | 28 | 17 | 1.6 ± 1.1 | 1 | 4 | 9 | 3 | S |
| *Enochrus testaceus (Fabr.)* | 10 | 9 | 1.1 ± 0.3 | 1 | 2 | 8 | 3 | S |
| *Helochares obscurus (O.F. Müll.)* | 410 | 144 | 2.8 ± 3.0 | 1 | 18 | 27 | 3 | S |
| *Helochares punctatus Sharp* | 2 | 2 | 1.0 ± 0.0 | 1 | 1 | 2 | 3 | S |
| *Hydrobius fuscipes (L.)* | 62 | 40 | 1.6 ± 0.9 | 1 | 5 | 15 | 3 | S |
| *Hydrochara caraboides (L.)* | 15 | 6 | 2.5 ± 2.8 | 1 | 8 | 5 | 4 | S |
| *Laccobius minutus (L.)* | 187 | 32 | 5.8 ± 8.3 | 1 | 34 | 11 | 2 | S |
| *Coelostoma orbiculare (Fabr.)* | 165 | 71 | 2.3 ± 2.6 | 1 | 18 | 25 | 2 | S |
| *Chaetarthria seminulum (Herbst. )* | 1 | 1 | 1.0 | 1 | 1 | 1 | 1 | S |
| *Cercyon convexiusculus Steph.* | 1 | 1 | 1.0 | 1 | 1 | 1 | 1 | S |
| *Cercyon litoralis (Marsh.)* | 1 | 1 | 1.0 | 1 | 1 | 1 | 2 | S |
| *^LC^ Cercyon tristis (Ill.)* | 2 | 2 | 1.0 ± 0.0 | 1 | 1 | 1 | 1 | S |
| *Cercyon nigriceps (Marsh.)* | 1 | 1 | 1.0 | 1 | 1 | 1 | 2 | S |
| *Limnebius parvulus (Herbst)* | 36 | 22 | 1.6 ± 1.4 | 1 | 5 | 12 | 1 | S |
| *Ochthebius minimus (Fabr.)* | 3 | 3 | 1.0 ± 0.0 | 1 | 1 | 3 | 1 | S |
| *Hydraena palustris Er.* | 5 | 3 | 1.7 ± 1.2 | 1 | 3 | 1 | 1 | S |
| Total | 10139 | 485 |  |  |  |  |  |  |
